# Diabetes and Frail Older Patients: Glycemic Control and Prescription Profile in Real Life

**DOI:** 10.3390/pharmacy9030115

**Published:** 2021-06-22

**Authors:** Anne-Sophie Mangé, Arnaud Pagès, Sandrine Sourdet, Philippe Cestac, Cécile McCambridge

**Affiliations:** 1 Department of Pharmacy, Toulouse University Hospital, UPS Toulouse III Paul Sabatier University, 31000 Toulouse, France; as.mange@ch-montauban.fr (A.-S.M.); cestac.p@chu-toulouse.fr (P.C.); mccambridge.c@chu-toulouse.fr (C.M.); 2 UMR 1027, Inserm, UPS Toulouse III Paul Sabatier University, 31000 Toulouse, France; 3 INSPIRE Project, Institute of Aging, Gérontopôle, Toulouse University Hospital, UPS Toulouse III Paul Sabatier University, 31000 Toulouse, France; 4 Geriatric Department, Toulouse University Hospital, UPS Toulouse III Paul Sabatier University, 31000 Toulouse, France; sourdet.s@chu-toulouse.fr

**Keywords:** diabetes mellitus, frail elderly, glycated hemoglobin A, glycemic control, medical overuse

## Abstract

(1) Background: The latest recommendations for diabetes management adapt the objectives of glycemic control to the frailty profile in older patients. The purpose of this study was to evaluate the proportion of older patients with diabetes whose treatment deviates from the recommendations. (2) Methods: This cross-sectional observational study was conducted in older adults with known diabetes who underwent an outpatient frailty assessment in 2016. Glycated hemoglobin (HbA1c) target is between 6% and 7% for nonfrail patients and between 7% and 8% for frail patients. Frailty was evaluated using the Fried criteria. Prescriptions of glucose-lowering drugs were analyzed based on explicit and implicit criteria. (3) Results: Of 110 people with diabetes with an average age of 81.7 years, 67.3% were frail. They had a mean HbA1c of 7.11%. Of these patients, 60.9% had at least one drug therapy problem in their diabetes management and 40.9% were potentially overtreated. The HbA1c distribution in relation to the targets varied depending on frailty status (*p* < 0.002), with overly strict control in frail patients (*p* < 0.001). (4) Conclusions: Glycemic control does not seem to be routinely adjusted to the health of frail patients. Several factors can lead to overtreatment of these patients.

## 1. Introduction

Experts are increasingly taking frailty and drug tolerance into account in making recommendations to guide the treatment of older adults [1,2]. Frailty is defined as a clinical syndrome reflecting a decline in physiological reserves [3,4]. Currently, the most widespread approach for assessing frailty is the Fried Frailty Index, which uses five criteria: weight loss, exhaustion, slow walking speed, weakness, and low levels of physical activity [3]. Patients are considered “robust” if their score is 0, “prefrail” if their score is 1 or 2, and “frail” if it is 3, 4, or 5. Frailty is a transient state, potentially reversible if the geriatric patient receives appropriate treatment [1,5].

Older patients with diabetes are particularly at risk of iatrogenic events due to the accumulation of age-specific functional deficits and disease progression, comorbidities, and ensuing treatments. Therapeutic optimization aimed at reducing this risk includes decreasing the number of drugs, taking the drugs’ pharmacokinetic properties into account, and reassessing glycemic targets based on the patient’s health. Overly strict glycemic control can result in hypoglycemia, increasing the risk of dementia and falls [6,7]. It was with this in mind that the therapeutic targets were adjusted. Several national and international recommendations set different targets for HbA1c depending on the level of frailty, allowing higher targets for frail patients [1,8,9,10,11,12]. Moreover, some glucose-lowering medications appear in lists of potentially inappropriate medications (PIMs) in older subjects because of their hypoglycemic properties, lack of proven efficacy, or lack of safety data [13].

The primary aim of this study was to assess the proportion of older people with diabetes whose treatment deviates from current recommendations. Additionally, we aimed to describe the nature of these deviations using the following items: HbA1c targets, PIMs, dosage adjustments to kidney function, contraindications, and hypoglycemia.

## 2. Materials and Methods

We performed a cross-sectional, observational, single-center study. We collected data from patients suffering from diabetes amongst those evaluated in 2016 at the Geriatric Frailty Clinic (GFC), an outpatient clinic of Toulouse University Hospital, France [14]. Data (sociodemographic data, medical history, and pharmaceutical treatments) were gathered for this study from the GFC software. Creatinine clearance was calculated using the Cockcroft formula. This study is reported in compliance with the STROBE guidelines [15].

These patients were referred to the GFC by their primary care physician to investigate whether they were frail or not, in order to provide them an individualized care plan [16]. For each patient, medication reconciliation was performed by the pharmacist and suggestions for therapeutic optimization were included in the individualized care plan. Furthermore, patients were asked for their approval before entering data for research purposes. The data were recorded in accordance with the French Data Protection Act and the General Data Protection Regulation (European Regulation No. 2016/679). The study was recorded in the register of the retrospective study of the Toulouse University Hospital (registration number: RnIPH 2021-75) and covered by MR-004 (CNIL number: 2206723 v 0). This study was approved by Toulouse University Hospital and confirmed to meet all the ethical requirements.

The glycemic balance was estimated by looking at a recent HbA1c assay (maximum 1 month old) or one performed during the geriatric assessment and compared with recommended targets. We defined HbA1c targets according to various guidelines [1,9,10] commonly used in France at the time of the study. These guidelines do not provide a lower limit, but morbidity/mortality studies such as the Action to Control Cardiovascular Risk in Diabetes (ACCORD) study and the Veterans Affairs Diabetes Trial (VADT) describe the risk of tight glycemic control (HbA1c < 6%) [17,18]. Therefore, we defined the following targets: between 6 and 7% for nonfrail patients and between 7 and 8% for frail patients. The missing data concerned only the HbA1c assay. These patients were excluded from the analysis for the glycemic balance. Frailty was evaluated using the Fried criteria [3]. Robust or prefrail patients with a Fried score of 0 to 2 were classified as “nonfrail”, while those with a score of ≥ 3 were considered “frail.”

The potential inappropriateness of glucose-lowering drug prescriptions was analyzed based on explicit criteria (European list of PIMs [13]) and implicit criteria (kidney function, HbA1c, history, and frailty level). As there are many explicit criteria tools available, we chose the EU(7)-PIM list instead of the Beers criteria because this list is more suitable to prescribing practices in Europe, and our study was conducted in France [19]. Prescriptions were classified as “potentially inappropriate” when there was at least one discrepancy with the reference standards. We categorized the drug therapy problems based on the criteria determined by the French Society of Clinical Pharmacy (SFPC): deviation from the reference standards (i.e., a PIM according to the European list), a contraindication, an overdose (dosage higher than the maximum recommended dose or HbA1c lower than identified target and dosage that could be decreased), an underdose (HbA1c higher than recommended target for which the dosage could be increased), a drug that is not indicated (HbA1c lower than identified target and treatment at the recommended minimum dosage or potentially harmful), an insufficiently treated indication (HbA1c higher than target requiring additional treatment), and occurrence of an adverse event [20]. We also considered the patient’s clinical geriatric assessment. We gathered proposals for therapeutic optimization of diabetes management by the pharmacist in consultation with the geriatrician.

Qualitative variables were described as numbers and percentages. The quantitative variables were calculated as means and standard deviations in the case of normal distribution, and as an interquartile interval (25th and 75th percentile of the distribution) if otherwise. The normality of the distribution was assessed by a graphical method and optionally by a Kolmogorov–Smirnov test.

To test the association between frailty and other qualitative variables of interest, we used the χ^2^ test if the validity conditions were met; otherwise, we used Fisher’s exact test. To compare the means between frail and nonfrail patients (i.e., robust or prefrail patients) (bilateral test), we used the Student’s *t* test if distribution was normal or the Wilcoxon signed-rank test if distribution was not normal. A difference was considered statistically significant for a *p* value of less than or equal to 0.05. The statistical analyses were performed using SAS 9.3 software (SAS Institute, Cary, NC, USA).

## 3. Results

### 3.1. Characteristics of Study Population

Of the 929 outpatients evaluated at the Toulouse GFC in 2016, 11.8% (*n* = 110) were known as suffering from diabetes. The general characteristics and treatment strategies for this population are described in Table 1.

According to the Fried criteria, 67.3% of the patients were frail. Polypharmacy (5 or more medications) affected 79.6% (*n* = 86) of patients. All of the patients in our study were taking at least one medication, with 2 to 20 drugs per patient (not including self-medication). Table A1 in Appendix A details the various glucose-lowering medication prescribed.

### 3.2. Analysis of Glucose-Lowering Drug Prescriptions’ Appropriateness

The drug therapy problems (DTPs) found in our analysis of diabetes management are described in Table 2. We calculated the subtotal corresponding to patients with at least 1 DTP without taking into account Acarbose and Liraglutide since these two criteria may be controversial.

Out of 110 patients, 67 (60.9%) had more than one DTP. The mean number of DTP for these 67 patients was 1.45. Of these patients, 40.9% (*n* = 45) were potentially overtreated, with at least one drug that was either not indicated and/or overdosed; 16.4% (*n* = 18) were insufficiently treated. Among patients with at least one DTP, one or more therapeutic optimization recommendations were made for 39 patients.

With regard to dose adjustment to renal function, we had the estimate of the Glomerular Filtration Rate (GFR) for each patient, as determined from serum creatinine using the Cockroft and Gault formula. The proportion of patients with a dosage that was not appropriate for their GFR was verified. Table A2 details the distribution of prescriptions at inappropriate dosages for impaired kidney function.

### 3.3. Analysis of Glycemic Balance

**Figure 1:**
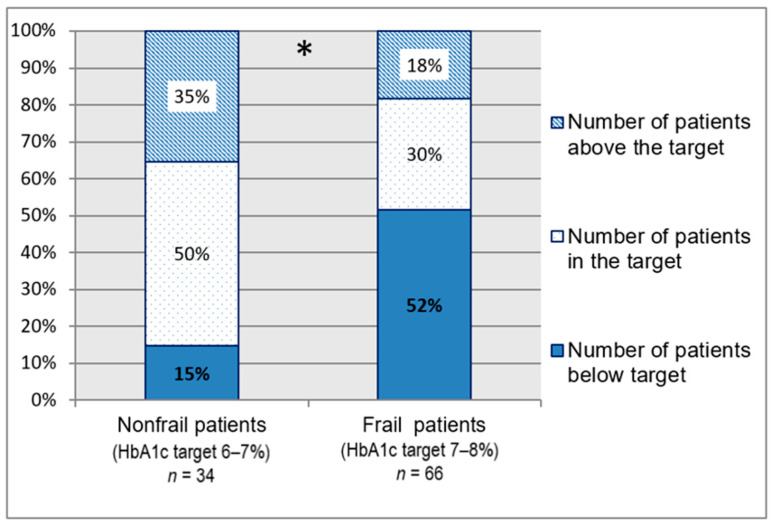
Patient distribution according to HbA1c targets (*n* = 100). HbA1c = glycated hemoglobin. Compliance with HbA1c targets varied significantly depending on whether the patients were frail or not; * *p* < 0.002. These data show that frail patients are more likely to be below the glycemic targets compared with nonfrail patients (*p* < 0.001). The HbA1c distribution based on the level of frailty is detailed in Figure A1. Glycated hemoglobin levels and glucose-lowering treatments based on frailty level are described in Table A3.

One hundred patients had a recent HbA1c assay (missing data: *n* = 10). The mean HbA1c calculated was 7.11% (± 1.11). Figure 1 shows the compliance with HbA1c targets based on frailty.

### 3.4. Hypoglycemic Episodes

Hypoglycemic episodes were suspected or confirmed in 9.1% of the patients (*n* = 10), with no significant difference between frail and nonfrail patients (*p* = 0.99). Of these patients, 7 were treated with insulin therapy, including one in combination with repaglinide; and 2 were treated with long-acting sulfonylureas. One patient developed hypoglycemia on monotherapy with repaglinide. Alongside these hypoglycemic episodes, we analyzed falls. Of the 23 patients with falls who were being treated “pharmacologically” and for whom a recent HbA1c was available, 47.8% (*n* = 11) had an HbA1c below the target. Of the patients with falls, one-third (*n* = 12) were taking a hypoglycemic agent (insulin, sulfonamide, or repaglinide).

## 4. Discussion

We noticed that diabetes management deviated from the recommendations for 60.9% of our study’s patients, and that 40.9% were potentially overtreated. We found that the glycemic objectives are not always suited to the health of the frailest patients, and the therapeutic streamlining allowed by the recommendations is not always applied. The overtreatment in frail elderly is a well-described topic in publications, including people with diabetes [21,22,23]. Our work describes various items of this overtreatment: the HbA1c targets adjusted for frailty, dosage adjustments to kidney function, and the concept of a PIM, which explains the high rate of DTP.

Regarding glucose lowering medications with a poor risk/benefit ratio, the EU(7)-PIM list mentions: fast insulin catch-up protocols, long-acting sulfonylureas, and acarbose. Because of their prolonged hypoglycemic effects, long-acting sulfonylureas are not recommended for the elderly, according to the international PIM list [13,24,25]. We found their prescription rate to be low (3.6%). Although the European list mentions glibenclamide and glimepiride as PIMs in older subjects, only the STOPP/START.v2 list additionally mentions gliclazide LM as inappropriate [13,25]. On the other hand, the European list proposes this one as a safer alternative. The Schernthaner study compared the safety of gliclazide LM with that of glimepiride. It found gliclazide to be significantly less hypoglycemic (3.7% vs. 8.9%; *p* < 0.003), with at least similar efficacy [26]. Given the literature, the place of sulfonylureas in the recommendations, and their efficacy, Gliclazide can be an alternative when other agents are not available for monitoring hypoglycemic risk. Concerning DPP4 inhibitors, the European list criticizes them for the lack of clinical safety data in older subjects. However, the latest French and European recommendations position them as second-line treatments [1,8,9,10]. Moreover, studies in older subjects are thought to favor their safe use, especially when individualized HbA1c targets are set [27,28]. Thus, we have deliberately not considered them as drug therapy problems.

Changes in the pharmacokinetic properties of the drugs in older subjects have to be considered in preventing drug-induced reactions. The OREDIA study was concerned with the treatment of older diabetes patients with kidney failure [22]. Of our patients with kidney failure, one-third had at least one drug for which the dosage should have been adjusted or replaced with an alternative treatment. As in OREDIA, we were able to conclude that impaired kidney function is not sufficiently taken into account in prescribing oral glucose-lowering drugs. Metformin was the drug most often found in these failures to adjust.

Although national recommendations did not recommend a lower limit for HbA1c targets, more recent national publications with European and international recommendations propose lower limits between 7% and 7.6%, particularly in frail patients [8,9,10]. Moreover, the targets proposed in the recent Sinclair’s international recommendations match with those used in our work [9]. This confirms the relevance of our targets. Several studies analyzed the relationship between mortality and glycemic control in older patients and described it as a U-shaped curve. The best-tolerated HbA1c levels are between 6% and 9%, with the lowest risk being at 7.5% [29,30,31]. Therefore, we defined the lower limit for HbA1c as 6% and 7% for nonfrail and frail patients, respectively. Thus, we found that a significantly larger percentage of frail patients had HbA1c levels below the target for their frailty status. This reinforces our previous observation regarding failure to adjust glycemic control for aging.

The GERODIAB and GUIDANCE studies concluded that glycemic control was too strict in older patients [23,32]. Subjects were enrolled in these studies before the new recommendations were published. Our study brings up a problem of compliance with the latest recommendations. This low adherence has also been discussed in other recent studies [33]. In order to improve it and reduce the overtreatment, the benefit of a multidisciplinary medication review combined with clinical pharmacist expertise is well described in both people with diabetes and elderly patients [34,35,36].

Hypoglycemic episodes increase the risk of falls, myocardial ischemia, impaired cognitive function, and mortality, and thus have to be prevented in elderly [37]. We identified 9.1% of patients as having had at least one hypoglycemic episode in the months preceding this study. The hypoglycemic histories we collected were patient-reported, so it is highly likely that the incidence of these events was underestimated. In older subjects, symptoms often go unnoticed, with vague and atypical symptoms, which complicates diagnosis after the fact. It is important to investigate any episode suggestive of hypoglycemia (dizziness, falls, etc.). Of the patients in our study who had fallen, almost one-third had glycemic control that was too strict as compared to the objectives. One-third were treated with at least one hypoglycemic agent. Although an older patient with diabetes may present with several reasons for a fall (peripheral neuropathy, muscle weakness, poor vision, etc.), the possibility of hypoglycemia should not be overlooked. The question of self-monitoring plasma glucose or stepping down treatment can be raised in these patients.

The main limitation of this study is the cross-sectional and monocentric design, which limits the generalizability of the results. We can also mention the limits for evaluating glycemic control in a single HbA1c assay, whose reliability may be influenced by certain factors impacting the turnover of red blood cells.

One of the strong points of our study is the use of the Fried score to identify frail patients. This allowed us to provide a documented estimate of the glycemic targets adjusted to the patient’s health. Moreover, the prescription analysis was done by pharmacists specialized in geriatrics and optimization recommendations were made in consultation with a multidisciplinary team. Finally, the use of implicit and explicit criteria to evaluate drug therapy problems increases the thoroughness of the analysis and the relevance of the recommendations.

## 5. Conclusions

This study provides an account of the prescription profile and glycemic control in Geriatric Frailty Clinic patients from Toulouse University Hospital in France. Several elements can lead to overtreatment in the frailest patients. The difficulty of managing older patients with diabetes lies in the search for a balance between abandoning treatment out of resignation and a fear of drug-induced reactions, and excessive intervention unsuited to the patients’ health status. The lack of a lower target in the national recommendations does not encourage deprescribing, especially in patients deemed to be stable. This raises the problem of reevaluating chronic diseases in stable but frail patients.

## Figures and Tables

**Table 1:** General population characteristics (*n* = 110).

Patient Characteristics	Statistics
Age (mean, standard deviation)	81.7 ± 6
Male (*n*, %) Female (*n*, %)	42 (38.2%) 68 (61.8%)
Weight (mean, standard deviation)	73.49 ± 14.6
**Kidney function (*n* = 108) ^a^**	
Serum creatinine (µmol/L) (mean, standard deviation)	101.1 ± 78.6
Creatinine clearance (mL/min) (mean, standard deviation)	55.8 ± 20.6
No CKD (*n*, %)	7 (6.5%)
Mild CKD: creatinine clearance between 60 and 90 mL/min (*n*, %)	35 (32.4%)
Moderate CKD: creatinine clearance between 30 and 60 mL/min (*n*, %)	55 (50.9%)
Severe CKD: creatinine clearance < 30 mL/min (*n*, %)	11 (10.2%)
**Frailty (*n* = 109) ^b^**	
Fried score (median, (Q25, Q75))	3 [2; 4]
**Fried frailty criteria ^b^**	
Weight loss (*n*, %)	22 (20.0%)
Feelings of exhaustion (*n*, %)	57 (52.3%)
Muscle weakness (*n*, %)	90 (82.6%)
Reduced walking speed (*n*, %)	64 (58.7%)
Sedentary lifestyle (*n*, %)	87 (79.1%)
**Falls (*n* = 107) ^c^**	
Yes (*n*, %)	36 (33.6%)
**Treatment**	
Number of medications prescribed ^d^ (mean, standard deviation)	8.2 ± 3.3
Number of glucose-lowering medication (mean, standard deviation)	1.4 ± 1.0
**Therapeutic strategy (*n*, %)**	*N* (%)
Lifestyle changes only	20 (18.2%)
Oral glucose-lowering drug only	51 (46.4%)
1 OGLD	32 (29.1%)
2 OGLDs	17 (15.5%)
≥3 OGLDs	2 (1.8%)
Oral glucose-lowering drug + Insulin	17 (15.5%)
1 OGLD + Insulin	9 (8.2%)
≥ 2 OGLDs + Insulin	8 (7.3%)
Insulin only	22 (20.0%)

Legend: CKD: chronic kidney disease; OGLD: Oral glucose-lowering drugs. ^a^ *n* = 2 for kidney function; ^b^ *n* = 1 for frailty score, feeling of exhaustion, reduced walking speed, muscle weakness; ^c^ *n* = 3 for falls; ^d^ *n* = 2 for number of prescribed medications.

**Table 2:** Drug therapy problems identified in diabetes management (*n* = 110).

Types of Problems	*n* = 110
**According to EU(7) PIM List**	
Long-acting sulphonylureas (glibenclamide, glimepiride)	4 (3.6%)
Acarbose	3 (2.7%)
Sliding-scale insulin	5 (4.6%)
**Contraindication**	**2 (1.8%)**
**Overdose**	
No adjustment to renal function	20 (18.2%)
Medication used at excessively high dose	10 (9.1%)
**Underdose**	**14 (12.7%)**
**Medication not indicated**	
Overly tight glycemic control relative to glycemic targets	22 (20.0%)
Pharmacological redundancy	1 (0.9%)
Liraglutide in a patient over 75 years	1 (0.9%)
**Indication not treated or insufficiently treated**	**4 (3.6%)**
**Hypoglycemia**	**10 (9.1%)**
**Subtotal:** patients with at least 1 DTP without taking into account Acarbose and Liraglutide	**66 (60.0%)**
**Total (patients with at least 1 DTP)**	**67 (60.9%)**

Legend: EU(7) PIM list: European list of potentially inappropriate medications; DTP: drug therapy problems (one patient can have several drug therapy problems). Dosage adjustments for patients with kidney failure (according to the Summaries of Product Characteristics available in 2016): Metformin: 1500 mg maximum per day if creatinine clearance is between 30 and 60 mL/min; contraindicated if creatinine clearance is <30 mL/ min). Sitagliptin: 50 mg maximum per day if creatinine clearance is between 30 and 50 mL/min; 25 mg maximum per day if creatinine clearance is <30 mL/ min). Acarbose: contraindicated if creatinine clearance is < 25 mL/min.

**Figure A1:**
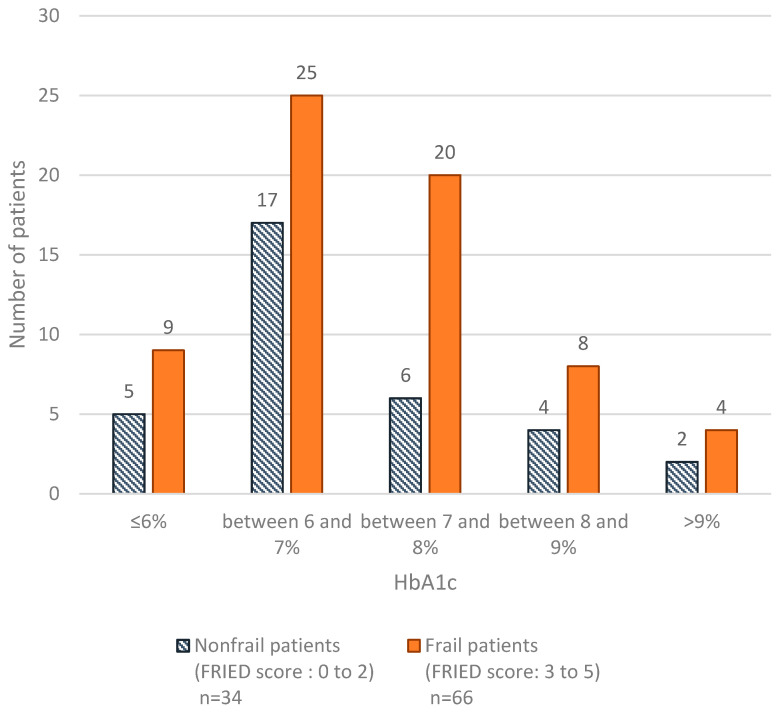
Distribution according to glycemic control and frailty (*n* = 100); HbA1c = glycated hemoglobin. Additional information—comparison of 3 groups: less than 7%, between 7 and 8%, more than 8% (chi-square test); *p*-value = 0.35.

## Data Availability

Research data are not shared.

## Appendix A

**Table A1:** Prescriptions of glucose-lowering drugs (90 patients).

Label	Number of Prescribed Medications (*n* = 151)	Average Daily Dosage
**Insulins and analogs**	**53 (35.1%)**	**IU**
Rapid-acting injectable insulins and analogs	14 (9.3%)	
Insulins and analogs with intermediate or long-acting action and rapid onset of action by injection	1 (0.7%)	
Long-acting injectable insulins and analogs	38 (25.2%)	
Insulin glargine	28	20.0
Insulin detemir	10	21.5
**Glucose-lowering** **drugs other than insulins**		**mg**
Biguanide	50 (33.1%)	1739.0
Sulfonylurea drugs	13 (8.6%)	
Glibenclamide	1	7.5
Gliclazide	9	85.6
Glimepiride	3	1.7
Alpha glucosidase inhibitors	3 (2.0%)	150.0
Dipeptidyl Peptidase 4 (DPP-4) inhibitors	19 (12.6%)	
Sitagliptin	15	96.4
Vildagliptin	4	75.0
Glucagon-Like-Peptide-1 (GLP-1) Analogs	2 (1.3%)	
Liraglutide	2	1.5
Repaglinide	11	2.6

**Table A2:** Distribution of potentially inappropriate medications (PIMs) related to kidney failure depending on frailty.

	Nonfrail Patients (FRIED Score: 0 to 2) *n* = 22	Frail Patients (FRIED Score: 3 to 5) *n* = 44	Total
Number of patients for whom all treatments are adjusted to kidney function	8 (36.4%)	25 (56.8%)	33 (50%)
Number of patients with at least 1 PIMs related to renal failure	8 ^a^ (36.4%)	13 ^b^ (29.5%)	21 (31.8%)
*Metformin*	6 (27.3%)	12 (27.3%)	18 (27.3%)
*Sitagliptin*	3 (13.6%)	5 (11.4%)	8 (12.1%)
*Acarbose*	1 (4.5%)	0 (0%)	1 (1.5%)

^a^ 2 Nonfrail patients with 2 PIMs (overuse of metformin and sitagliptin); ^b^ 4 frail patients with 2 PIMs (overuse of metformin and sitagliptin). Dosage adjustments for patients with kidney failure (according to the Summaries of Product Characteristics available in 2016): Metformin: 1500 mg maximum per day if creatinine clearance is between 30 and 60 mL/min; contraindicated if creatinine clearance is <30 mL/ min). Sitagliptin: 50 mg maximum per day if creatinine clearance is between 30 and 50 mL/min; 25 mg maximum per day if creatinine clearance is <30 mL/ min). Acarbose: contraindicated if creatinine clearance is < 25 mL/min.

**Table A3:** Description of glycemic control and prescription profile of patients according to their frailty phenotype.

	Nonfrail Patients (FRIED Score: 0 to 2)	Frail Patients (FRIED Score: 3 to 5)	*p* Value
**HbA1c level**	***n* = 34**	***n* = 66**	
≤7%	22 (64.7%)	34 (51.5%)	0.35 *
between 7% and 8%	6 (17.6%)	20 (30.3%)	
>8%	6 (17.6%)	12 (18.2%)	
**Drugs**	***n* = 36**	***n* = 74**	
Number of glucose-lowering drugs	1.3 ± 1.0	1.4 ± 1.0	0.79 ^#^
No drugs	7 (19.4%)	13 (17.6%)	0.66 *
OGLDs only	19 (52.8%)	32 (43.2%)	
Insulin	5 (13.9%)	17 (23.0%)	
OGLDs + Insulin	5 (13.9%)	12 (16.2%)	

HbA1c = glycated hemoglobin; OGLDs = oral glucose-lowering drugs, * chi-square test, ^#^ Wilcoxon test.
